# *Ganoderma lucidum* aqueous extract inducing PHGPx to inhibite membrane lipid hydroperoxides and regulate oxidative stress based on single-cell animal transcriptome

**DOI:** 10.1038/s41598-022-06985-z

**Published:** 2022-02-24

**Authors:** Wenqiao Ding, Xueying Zhang, Xiaoyu Yin, Qing Zhang, Ying Wang, Changhong Guo, Ying Chen

**Affiliations:** 1 Key Laboratory of Biodiversity of Aquatic Organisms, Harbin Normal University, Harbin, 150025 China; 2 College of Biology and Food Engineering, Jilin Institute of Chemical Technology, Jilin, 132022 China; 3 School of Civil and Environmental Engineering, Harbin Institute of Technology (Shenzhen), Shenzhen, 518055 China; 4 Key Laboratory of Molecular Cytogenetics and Genetic Breeding of Heilongjiang Province, College of Life Science and Technology, Harbin Normal University, Harbin, 150025 China

**Keywords:** Bioinformatics, Cytological techniques, Nutrition

## Abstract

In this study, the single-cell eukaryotic model organism *Tetrahymena thermophila* was used as an experimental material to reveal the anti-aging mechanism of *Ganoderma lucidum* aqueous extract. After treatment with the *G. lucidum* aqueous extract, the logarithmic phase was extended, and the maximum density of *T. thermophila* increased to 5.5 × 10^4^ cells/mL. The aqueous extract was more effective than the main active monomers of *G. lucidum*. The membrane integrity in the cell including mitochondria and nucleus appeared improvement after treatment with the *G. lucidum* aqueous extract, which observed by ammonia silver staining and transmission electron microscopy. Gene Ontology (GO) functional enrichment of the differentially expressed genes in transcriptome showed that the *G. lucidum* aqueous extract promoted the biological metabolic process of membrane components. According to Kyoto Encyclopedia of Genes and Genomes (KEGG), the glutathione metabolism process was enhanced in both growth phases. Protein–protein interaction (PPI) network analysis illustrated that phospholipid hydroperoxide glutathione peroxidase (PHGPx) played a key role in the anti-aging mechanism. The results suggested that *G. lucidum* aqueous extract improved the GPX activity as well as reduced the malondialdehyde content and cell damage. More importantly, the expression of PHGPx was promoted to reduce the oxidation degree of the membrane lipids and enhance the integrity of the membrane to achieve anti-aging effects.

## Introduction

As early as 100 BC, Shennong Materia Medica has classically recorded that *Ganoderma lucidum* can enhance body immunity and prolong lifespan^1^. Modern medical researches have proved that *G lucidum* was safe, tolerable and free of toxic effects^2,3^. Some studies have focused on *Ganoderma.* polysaccharide and proved it was safe and non-toxic for animal, which is very helpful for clinical application^4–6^. *G. lucidum* and its active substances have obvious protective effects against lipid peroxidation in the brain, heart, liver, gastrointestinal tract, kidney and other important organs^7,8^.These protective effects were mainly reflected in the enhancement of antioxidant enzyme activities^9^. Those reactive oxygen species (ROS) scavenging enzymes can maintain the redox balance in the body, participate in the body's defence response to oxygen stress, and resist external oxidative stress^10,11^. The fruiting body of *G. lucidum* contains selenium^12^, which helps to enhance the levels of selenium cysteine and thus enhance the activity of glutathione peroxidase (GPX). GPX plays a prominent role in the oxidation and signal transduction of hydrogen peroxide (H_2_O_2_) and can directly regulate ROS^13^. According to whether its catalytic site is selenocysteine (Sec) or cysteine (Cys), GPX can be divided into selenium-dependent GPX and selenium-independent GPX^13,14^. The effect of *G. lucidum* on the GPX protein family has rarely been reported, and the substrates of *G. lucidum* and its mechanism of regulating GPX expression are not fully understood. This would hinder the development of *G. lucidum* and its products for clinical use.

In this experiment, *Tetrahymena thermophila* was used as the experimental material. *T. thermophila* is a free-living ciliate that exists widely in global aquatic ecosystems and is similar to metazoan cells in structure and functional complexity^15^. The discovery of ribozyme^16^ and telomerase^17^ in *Tetrahymena* gave a major boost in anti-aging mechanism research, which were rewarded two Nobel Prizes^18^. Because of its short growth cycle, easy culture and clear genetic background^19^, *T. thermophila* could provide dual research in vivo and in vitro in one experimental design. *T. thermophila* has been successfully used in drug screening and pharmacological mechanisms^20^. More importantly, information of the molecular evolution of the *T. thermophila* glutathione peroxidase family has been uncovered^19–22^. The information profile of *T. thermophila* GPX genes can be obtained from the *Tetrahymena* Functional Genomics Database (TetraFGD) (http://tfgd.ihb.ac.cn/) and TGD (http://www.ciliate.org)^22^. Ten of the twelve putative GPX have been identified as phospholipid hydroperoxide glutathione peroxidase (PHGPx) in the *Tetrahymena* genome comparative database. PHGPx, a selenium dependent GPX, can specifically reduce phospholipid hydroperoxide to protect lecithin liposomes and biofilms from oxidative damage^23^. PHGPx in *T. thermophila* has an average molecular mass of approximately 21.7 kDa^22^, which is similar to the molecular mass (20–22 kDa) of the PHGPx monomer protein described in mammals^24^. Moreover, a large number of reports showed that the GPX expression of *T. thermophila* was quantitatively different and dependent on the stress source (oxidant, apoptosis inducer or metal)^21,22^ and exposure time^20–24^.

By comparing the effects of the aqueous extract and main monomers of *G. lucidum* on growth curve and maximum density of *T. thermophila*, this study obtained the best anti-aging *G. lucidum* extract. The anti-aging mechanism of *G. lucidum*, especially the determination of the action type of the GPX family, was investigated at the molecular, cellular and individual levels. The results of this study are expected to contribute to clinical application of *G. lucidum* products.

## Materials and methods

### *Ganoderma lucidum* aqueous extract and monomers

Referring to the report of Cuong^25^, 20 g of dried *G. lucidum* (Changbai Mountain Senbao Specialty Store, Jilin) was ground and extracted in 1000 mL of ddH_2_O. The aqueous extract was collected by centrifugation, concentrated to 100 mL by vacuum drying and stored at − 20 °C until use.

*G. lucidum* polysaccharide, Ganoderic Acid A*,* Ganoderal A and *G. lucidum* ergosterol were purchased from Chengdu Must Biotechnology Co., Ltd. These three standards were dissolved in methanol according to the uniform design and added to SPP medium.

### Cell culture and drug treatment

*Tetrahymena thermophila* (SB210) was provided by the Institute of Hydrobiology, Chinese Academy of Sciences, Wuhan, PR China. *T. thermophila* was cultured in a 28 °C incubator. The SPP medium contained 2% (w/v) proteose peptone, 0.1% yeast extract (Oxoid), 0.2% glucose, and 0.003% sequestrene. According to the uniform design (Supplementary Tables S1 and S2), *G. lucidum* aqueous extract, *G. lucidum* polysaccharide, Ganoderic Acid A*,* Ganoderal A and *G. lucidum* ergosterol were added to the culture medium. The control group was replaced by the same volume of double distilled water, with 3 parallel samples in each group. After entering the logarithmic phase, samples were taken every 2 h until the decline phase. The density of *T. thermophila* was counted by blood cell counting board, and the relationship between the density of *T. thermophila* and time was drawn.

### Transcriptional analysis

The experiment was carried out with a 24-well plate, and each well was made up of 900 μL SPP. In the experimental group, 100 μL of 200 mg/mL *G. lucidum* aqueous extract was added. The control group was treated with 100 μL ddH_2_O instead. Three parallel samples were set in each group. The control group and experimental group were both in logarithmic phase (20 h), and the decline phase (27 h) was collected for the transcriptomic analysis. Each group was biologically duplicated, and all samples were subjected to a whole-transcript microarray assay (Biomarker Technologies, Beijing, China). The gene expression profile was detected by an Affymetrix 3′ IVT Expression Array. To reduce the effect of the expression of value genes, RPKM ≥ 5 and FC (Fold Change) ≥ 2 were used as the criteria to screen differentially expressed genes (DEGs). KOBAS was used to realize the path analysis of Gene Ontology (GO) and Kyoto Encyclopedia of Genes and Genomes (KEGG). The enrichment pathway was determined by the corrected *P*-adj value ≤ 0.05. The transcriptome results were verified using quantitative reverse transcription PCR (RT-qPCR) (Method see Supplementary file). Then, the protein–protein interaction (PPI) network was established based on the STRING database, and the central gene was screened by using the CytoHubba to identify the central node in Cytoscape software.

### Silver staining and transmission electron microscopy (TEM) observation

The procedure of silver staining was according to Foissner’s method^26^. *T. thermophila* was observed under an oil microscope. The ultrastructure of *T. thermophila* was observed by TEM. The cells treated with *G. lucidum* aqueous extract and the control group were collected by centrifugation at 6000×*g* for 2 min. Cells were prefixed in 2.5% glutaraldehyde and maintained at 4 °C for 8 h. The samples were washed with PBS (pH 7.5) three times, centrifuged at 3000×*g* for 5 min, and then fixed with 1% osmium tetraoxide (4 h). Thereafter, dehydration was performed using gradient acetone of 15% to 100%. Then, the cells were embedded in Embed 812 (TAAB) low viscosity resin. After curing, ultrathin sections (60–80 nm) were cut by an ultrathin slicer. The ultrastructure was observed by transmission electron microscope (JEOL JEM-1010, Japan).

### Determination of cell damage

Intracellular ROS was detected by the sensitive fluorescent probe 2′,7′-dichlorodihydrofluorescein diacetate (DCFH-DA)^27^. The activity of malondialdehyde (MDA) was determined by commercial kits provided by Nanjing Jiancheng Bioengineering Institute (Nanjing, Jiangsu, China). The method for determining the ability to eliminate hydroxyl radicals (·OH) was as described by Takemura^28^. The ability of the *G. lucidum* aqueous extract to eliminate ·OH free radical was expressed as the optical density of the experimental group minus the optical density of the control group.

### PHGPx activity

Nicotinamide adenine dinucleotide phosphate (NADPH)/NADP^+^ ratio and glutathione/glutathione disulfide (GSH/GSSG) ratio were determined using NADP^+^/NADPH Quantification Kit (S0179, Beyotime), GSH/GSSG Assay Kit (S0053, Beyotime). All these assays were performed according to the manufacturer’s protocol.

## Results

### The effects of *G. lucidum* aqueous extract and *G. lucidum* monomers on the growth of *T. thermophila*

As previous studies^7^, *G. lucidum* polysaccharide was the main component of *G. lucidum* aqueous extract in this experiment according to spectrophotometer identifying (Supplementary Fig. S1). The active substances of *G. lucidum* aqueous extract were also analyzed by GC–MS. The total ion diagram of the *G. lucidum* aqueous extract showed that 11 components existed in the aqueous extract (Supplementary Fig. S2). Among the compounds of the *G. lucidum* aqueous extract (Supplementary Tables S3 and S4), *G. lucidum* acid A, *Ganoderal* A and *G. lucidum* ergosterol were selected according to their retention time and matching degree.

**Figure 1:**
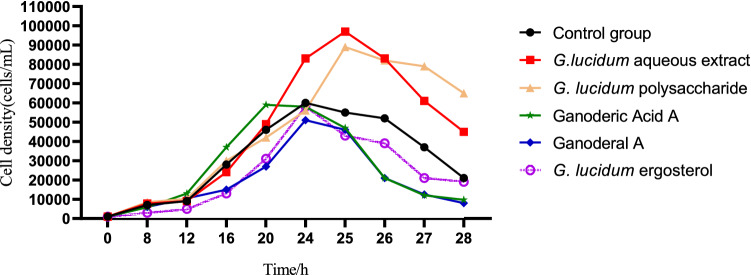
Effects of *G. lucidum* aqueous extract and *G. lucidum* monomers on the growth of *T. thermophila*.

In order to compare the effects of *G. lucidum* aqueous extrac and these main monomers on the growth of *T. thermophila*, the *G. lucidum* aqueous extract and *G. lucidum* monomers solution were added to the culture medium of *T. thermophila*. The results were shown in Fig. 1. The maximum density of the control group was 6 × 10^4^ cells/mL at 24 h. The cell density of the *G. lucidum* aqueous extract group from 12 to 20 h was almost the same as that of the control group, but from 20 to 25 h it was significantly higher than the cell density of the control group. Simultaneously, the logarithmic phase was prolonged by 1 h, the maximum density was 1.15 × 10^5^ cells/mL. Although the maximum density of the *G. lucidum* polysaccharide group was higher than that of the control group by 9.7 × 10^4^ cells/mL, it was lower than the *G. lucidum* aqueous extract group. The maximum density of *G. lucidum* acid A, *Ganoderal* A and *G. lucidum* ergosterol did not exceed that of the control group, which showed that the alcohol solutes of these monomers were not conducive to the growth of *T. thermophila*. It could be concluded that the *G. lucidum* aqueous extract containing a variety of active substances can promote the growth of *T. thermophila* more than its monomers.

### Anti-aging effect of the *G. lucidum* aqueous extract

**Figure 2:**
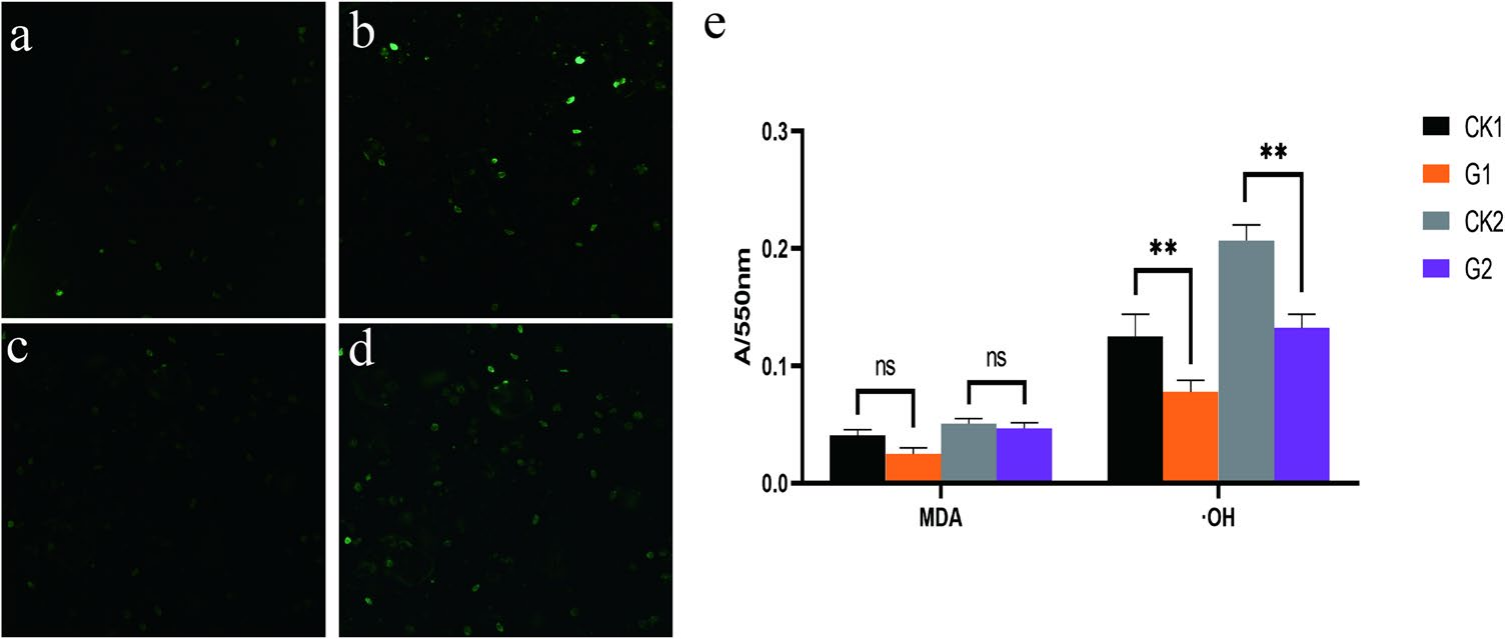
Effect of *G. lucidum* aqueous extract on ROS, MDA and ·OH in *T. thermophila*. (**a**–**d**, ×100). (**a**) ROS fluorescence in the logarithmic phase of the control group. (**b**) ROS fluorescence showed in the control group during the decline phase. (**c**) ROS fluorescence in the logarithmic phase of the experimental group applicated with *G. lucidum* aqueous extract. (**d**) ROS fluorescence in the decline phase of the experimental group applicated with *G. lucidum* aqueous extract. (**e**) The accumulation of MDA volume and ·OH in logarithmic and decline phases in the control and experimental groups. Symbols: CK1, the logarithmic phase of the control group. CK2, the decline phases of the control group. G1, the logarithmic phase of the experience group. G2, the decline phase of the experience group. *p < 0.05, **p < 0.001, ns, not significant difference.

With culture time increasing, the light intensity of ROS in the decline phase (Fig. 2b) was higher than that in the logarithmic phase (Fig. 2a). After adding the *G. lucidum* aqueous extract, although the fluorescence intensity of the decline phase (Fig. 2d) was still enhanced compared with that of the logarithmic phase (Fig. 2c), it was significantly lower than that of the control group. The MDA content in the control group was also higher in the decline phase than in the logarithmic phase (Fig. 2e). Meanwhile the scavenging ability of ·OH in the decline phase was lower than that in the logarithmic phase (Fig. 2e). The balance between oxidation and reduction was lost and the body aged. After adding the *G. lucidum* aqueous extract, the MDA content was lower than that in the control group (left column in Fig. 2e), and the amount of ·OH scavenged by cells in the logarithmic phase and decline phase was higher than that in the control group (right column in Fig. 2e). The growth of *T. thermophila* can be compared with the aging process of multicellular organisms through its cellular damage. As ROS and MDA accumulating, growth was inhibited and the density of *T. thermophila* was reduced. The addition of *G. lucidum* aqueous extract could maintain the redox balance in *T. thermophila* and achieve anti-aging effects.

### Analysis of differentially expressed genes and verification by RT-qPCR

Compared with the control group, the expression of 11,519 genes in the logarithmic phase of *T. thermophila* significantly changed after adding *G. lucidum* aqueous extract to SPP. Among them, 6201 genes were up-regulated and 5318 genes were down-regulated. The expression of 8772 genes changed significantly during the decline phase, among which 4721 genes were up-regulated and 4051 genes were down-regulated. In previous studies in our laboratory, the anti-aging effect of *G. lucidum* on *Stylonychia* was reflected in the antioxidant index, so 10 genes (Supplementary Table S5) related to antioxidants were randomly selected from among the DEGs, and the accuracy of the RNA-seq data was verified by RT-qPCR. The expression patterns of these 10 genes in the logarithmic phase and decline phase were consistent with the trend of the RNA-seq data (Supplementary Fig. S3). The result indicated that the RNA-seq data was reliable and could be used for subsequent analysis.

### GO enrichment and KEGG pathway analyses

**Figure 3:**
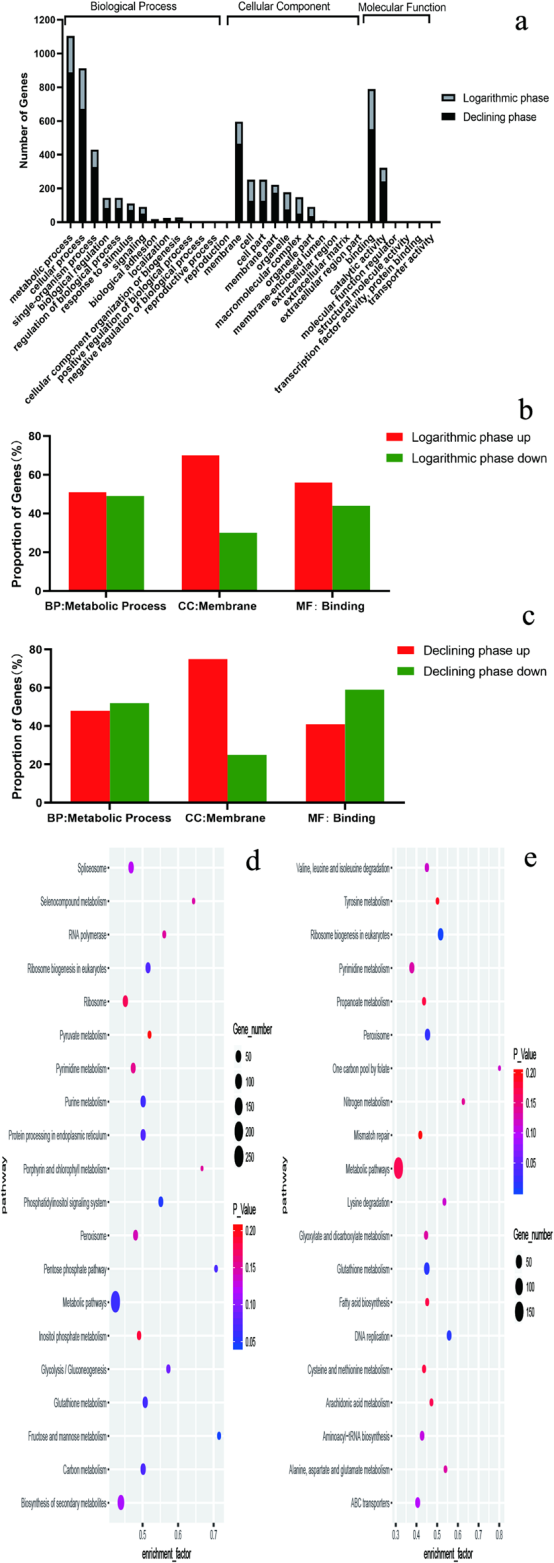
GO enrichment analysis and KEGG pathway enrichment. (**a**) GO enrichment analysis in logarithmic phase and decline phase. (**b**) The proportion of up or down regulated genes in BP, MF and CC in logarithmic phase. (**c**) The proportion of up or down regulated genes in BP, MF and CC in decline phase. (**d**) KEGG enrichment in logarithmic phase. (**e**) KEGG enrichment of different genes in decline phase.

The DEGs in the logarithmic phase were involved in 14 biological processes (BP) with 6 types of molecular functions (MF) and they existed in 11 cell components (CC) (Fig. 3a). The DEGs in the decline phase were involved in 12 BP with 4 kinds of MF and they existed in 12 CC. The most involved BP in the two phases was the metabolic process, MF was binding, and CC was membrane. Among the metabolic process, binding and membrane, the up-regulated genes accounted for 70% in the logarithmic phase (Fig. 3b) and 75% in the decline phase (Fig. 3c). These results show that the *G. lucidum* aqueous extract could promote the maintenance of cell membrane integrity in both phases.

KEGG pathway enrichment analysis was performed to further analyze the DEGs between the two phases. In the logarithmic phase, 94 pathways were concentrated, among which 68% of the total genes were involved in metabolism. In the decline phase, 91 pathways were enriched, among which the metabolism was still the largest pathway, accounting for 68%. The top 20 pathways in the logarithmic phase (Fig. 3d) and decline phase (Fig. 3e) were used to draw a bubble chart and showed that regardless of the significance or the number of genes involved, the effect of the *G. lucidum* aqueous extract primarily focused on the glutathione metabolism pathway. In the logarithmic phase, 38 differentially expressed genes were enriched in this pathway, accounting for 45%. In the decline phase, 43 differentially expressed genes were enriched in this pathway, accounting for 50%**.**

### The protein–protein interaction (PPI) networks of DEGs

The elucidation of DEGs in the glutathione metabolism pathway and insight into their interactions with other proteins will help to further explore the potential regulatory mechanism of *G. lucidum* aqueous extract.

**Figure 4:**
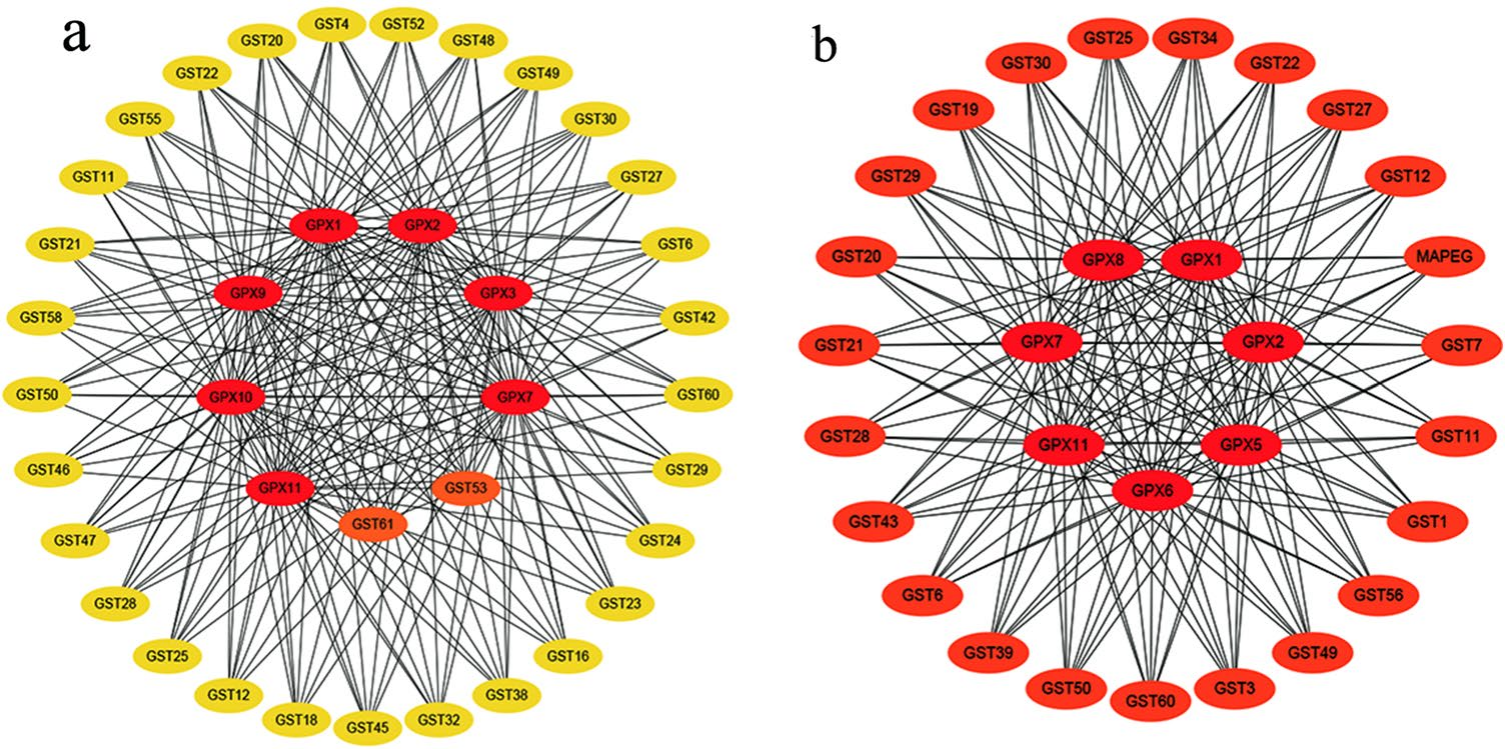
PPI mapping of DEGs in the glutathione-metabolism pathway. (**a**) Logarithmic phase. (**b**) Decline phase.

Both the logarithmic phase (Fig. 4a) and decline phases (Fig. 4b) had the most important effects on GPX1, GPX2, GPX7 and GPX11. Through the query of the TetraFGD database, these GPX enzymes belonged to PHGPx (Supplementary Table S6). Therefore, antioxidant enzymes, especially PHGPx, play an important role in promoting the growth of *T. thermophila* via the inducing of *G. lucidum* aqueous extract.

### PHGPx activity and function verification

**Figure 5:**
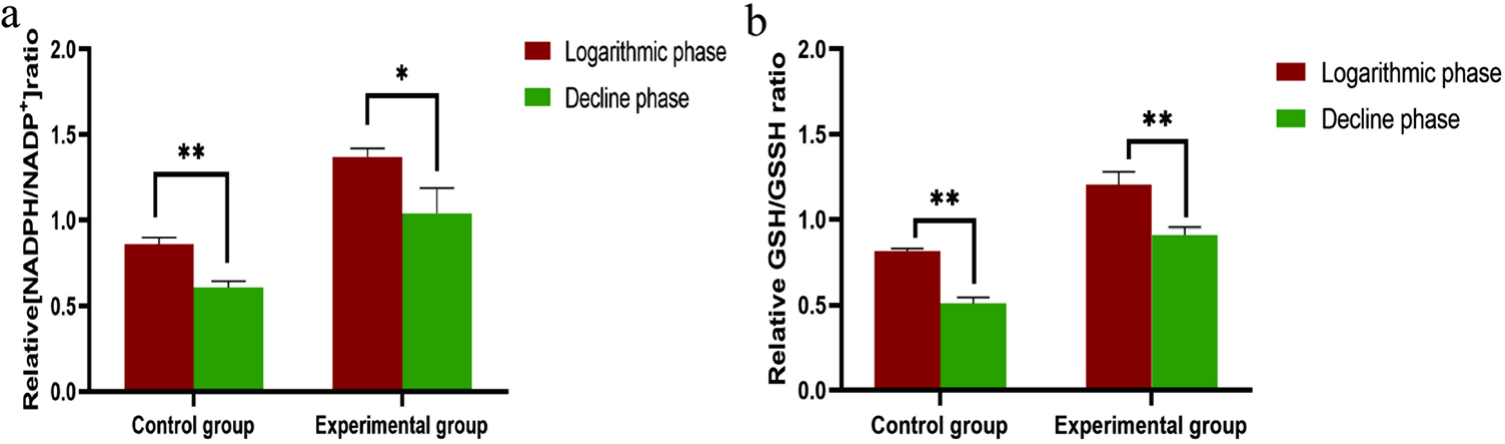
The activity of PHGPx in the logarithmic and decline phases. (**a**) The NADPH/ NADP^+^ ratio of *T. tetrahymena* in the two phases between control and experimental groups (**b**) The GSH/GSSH ratio of T. tetrahymena in the two phases between control and experimental groups Symbols: *p < 0.05, **p < 0.001, ns, not significant difference.

The activity of PHGPx refers to the rate of NADPH absorption decrease^29^. The NADPH/NADP^+^ value in the decline phase of the control group was lower than that in the logarithmic phase (Fig. 5a). The result suggested that the activity of PHGPx decreased with the increase of culture time. After adding *G. lucidum* aqueous extract, the NADPH/NADP^+^ value in the decline phase was still lower than that in the logarithmic phase, but higher than both phases of the control group. This result indicated that the *G. lucidum* aqueous extract could promote the activity of PHGPx.

PHGPx can specifically catalyze the reduction of phospholipid hydroperoxide by reduced glutathione (GSH), so as to convert toxic lipid peroxide (PL-PUFA-OOH) to non-toxic polyunsaturated fatty acids (PL-PUFA-OH) efficiently^30^. The change of GSH/GSSH ratio can reflect the change of PL-PUFA-OOH and PL-PUFA-OH Due to the decrease of PHGPx activity (Fig. 5a), the ratio of GSH/GSSH in the control group also decreased with the increase of culture time (Fig. 5b). However, after adding the *G. lucidum* aqueous extract, the ratio of GSH/GSSH was increased in logarithmic phase and decline phase. The Intracellular toxic PL-PUFA-OOH in experimental group was transformed into non-toxic PL-PUFA-OH, which was more than that of the control group.

### The observation of microscopic morphology and ultrastructure

**Figure 6:**
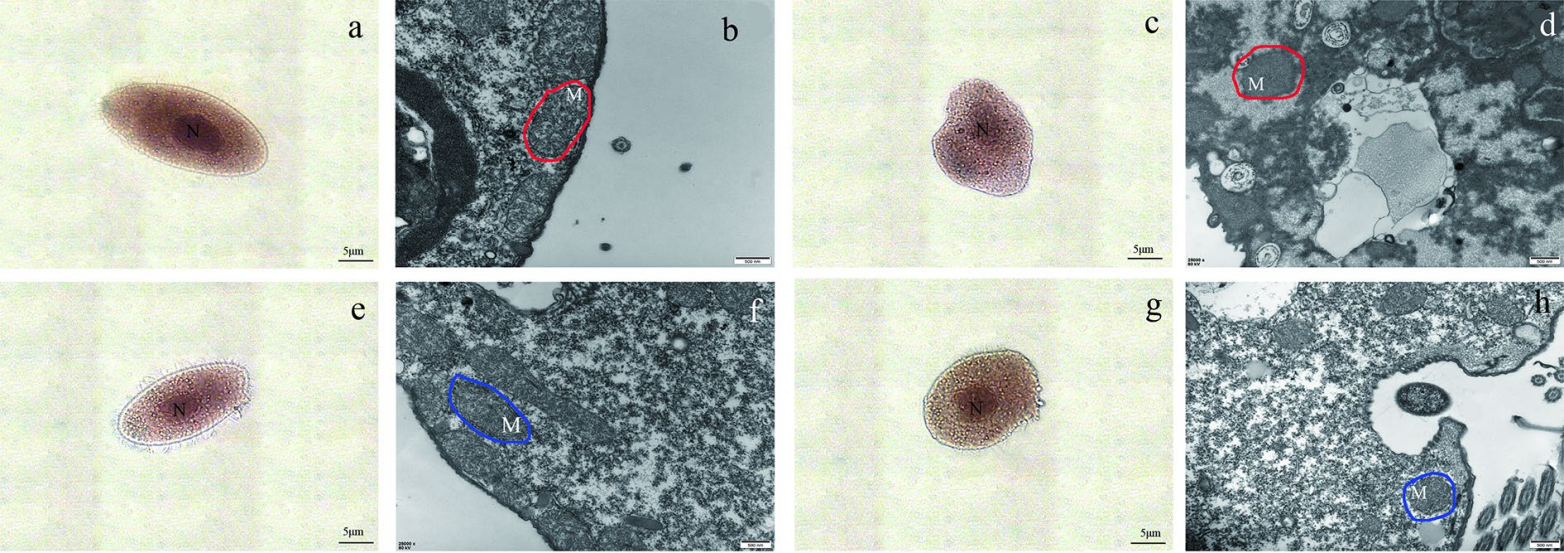
The observation of micromorphology and ultrastructure in control cells and experimental cells. (**a**) The ammonia silver staining of control group in the logarithmic phase. (**b**) The TEM observation of control group in the logarithmic phase. Mitochondria were circled by a red coil. (**c**) The ammonia silver staining of control group in the decline phase. (**d**) The TEM observation of control group in the decline phase. Mitochondria were circled by a red coil. (**e**) The ammonia silver staining of experimental group in the logarithmic phase. (**f**) The TEM observation of experimental group in the logarithmic phase. Mitochondria were circled by a blue coil. (**g**) The ammonia silver staining of experimental group in the decline phase. (**h**) The TEM observation of experimental group in the decline phase. Mitochondria were circled by a blue coil.

The protective effect of *G. lucidum* aqueous extract on the membrane was visualized by ammonia silver staining and TEM. By comparing logarithmic phase of control group (Fig. 6a) and decline phase of control group (Fig. 6c), it was found that the cell morphology of *T. thermophila* was seriously deformed with the extension of culture time, and the nucleus was blurred, indicating that the cells appeared to be in an aging state. However, in *G. lucidum* aqueous extract group, the morphology and integrity of the cells were well protected in the logarithmic phase (Fig. 6e) and the decline phase (Fig. 6g). Ultrastructural changes in the cells were observed by TEM. The ridge and contour of mitochondrial were blurred in decline phase (Fig. 6d) compared with logarithmic phase (Fig. 6b) in the control group. Mitochondria decay increased with the extension of culture time. The addition of *G. lucidum* aqueous extract improved the morphology of the mitochondria in the logarithmic phase (Fig. 6f) and decline phase (Fig. 6h). *G. lucidum* aqueous extract guaranteed the function of mitochondria, which is closely related to the integrity of the membrane. It should be noted that in the logarithmic phase, the mitochondria in the experimental group showed morphological changes (Fig. 6f, h), but the integrity of the membrane was not affected.

## Discussion

The application of *G.lucidum* extracts has a long history both in traditional Chinese medicine and East Asia. A large number of studies have shown their efficacy. Previous reports found that *G.lucidum* aqueous extract contained not only *G. lucidum* polysaccharides but also *G. lucidum* triterpenoids. According to previous reports, *Ganoderma* acid*, Ganoderma* aldehyde and *Ganoderma* ergosterol are the main ingredients of *G. lucidum* triterpene^31^. They act different repair function for cell (Table 1).

**Table 1:** Anti-aging researches of *G. lucidum* aqueous extract and main active monomers.

Extraction	Previous work
*G. lucidum* aqueous extract	Contains several bioactive phytochemicals such as polysaccharides, nucleosides, alkaloids, coumarin, ergosterols, ganoderic acids, lactones, mannitol, organic germanium, triterpenoids, unsaturated fatty acids^1,32^
	Increase radical scavenging activity and ferric reducing antioxidant power^1,32,33^
	Adjuvant treatment of neurological diseases^1,9,24,34,35^
*G. lucidum* polysaccharide	Antioxidant, immunomodulatory, antineurodegenerative and antidiabetic activities^1,36^
Ganoderma acid	Interact with membrane receptors mainly, receptor tyrosine kinase (RTKs). Ganoderic acid interacts and modulates the signaling network in IR, IGFR-1, IGFR-2, VEGFR-1, VEFGR-2, and EGFR in cancer signaling pathways. It primarily targets NF-κB, RAS-MAPK, PI3K/Akt/mTOR, and cell cycle resulting in apoptosis^37^
Ganoderma aldehyde	Inhibitted the growth of liver cancer PLC/PRF/5 and KB cells^38^
*G. lucidum* ergosterol	To ensure cell viability, membrane fluidity, membrane binding enzyme activity, membrane integrity, and cellular material transport plays an important role^39^

*Ganoderma lucidum* aqueous extract contains a variety of active substances, integrating the functions of the other four active substances, can not only resist oxidation, but also keep membraneintegrity. In this study, the growth-promoting of the *G. lucidum* aqueous extract on *T. thermophila* verified this inclusion relationship. Through the analysis of *G. lucidum* aqueous extract by total-ion flow chromatography (Supplementary Fig. S2), it could be found that the dissolution time of various active substances was different. So the *G. lucidum* aqueous extract becomes a complex organic solvent with the extension of the boiling time. We think that the active substances in the *G. lucidum* aqueous extract might act at different times, resulting in a synergy. This should be a further study in the future.

The analysis of *T. thermophila* transcriptome explored the effect of *G. lucidum* aqueous extract on gene expression during growth. After treatment with *G. lucidum* aqueous extract, PHGPx appeared a key influence in cell density and generation time. This result confirmed that the *G. lucidum* aqueous extract acted on the selenium-dependent type of GPX family. PHGPx is a multifunctional seleno-protein which widely distributed in the body that can not only participate in antioxidant reactions but also directly reduce the lipids of membrane to protect the integrity of the membrane system^40^. Compared with other GPXs, PHGPx has a smaller volume and greater hydrophobicity. Many reports have demonstrated that PHGPx overexpression in cells can resist cell response to endogenous (e.g., aging)^41^ and exogenous (e.g., environmental) peroxidation damage^42–45^. PHGPx is the only enzyme that can directly reduce the PL-PUFA-OOH of the membrane into the corresponding hydroxyl compounds, thereby enabling the termination of peroxidation and protecting the biological membrane from peroxidation damage^46,47^. The increase in the content of PL-PUFA-OOH was the reason for the damage of the cell membrane in the control group during the decline phase, which became a vicious cycle and accelerated cell senescence. In this study, the contents of ROS and MDA (Fig. 2) of *T. thermophila* in the control group increased with the increase of culture time. The membrane appeared serious deformation in the decline phase through the morphological observation. However, when *G. lucidum* aqueous extract was added, the expression of PHGPx was up-regulated. Thus, PL-PUFA-OOH on the membrane was transformed into nontoxic PL-PUFA-OH, and the stability of the membrane structure is enhanced both in the logarithmic phase and decline phase. According to the observation of the membrane by ammonia silver staining and TEM, it could be concluded that the *G. lucidum* aqueous extract penetrated the cell membrane during the growth process. This repair effect on the cell membrane could induce the active substances of the *G. lucidum* aqueous extract to enter the cytoplasm and protect the membrane structure of the nucleus and mitochondria to promote the metabolism of *T. thermophila* and achieve anti-aging effects. As the results of the KEGG enrichment show, a large number of differential genes are concentrated in the metabolic pathways. These results indicated that the *G. lucidum* aqueous extract was beneficial to the metabolism of glutathione and the elimination of toxic peroxides produced by cell aging.

**Figure 7:**
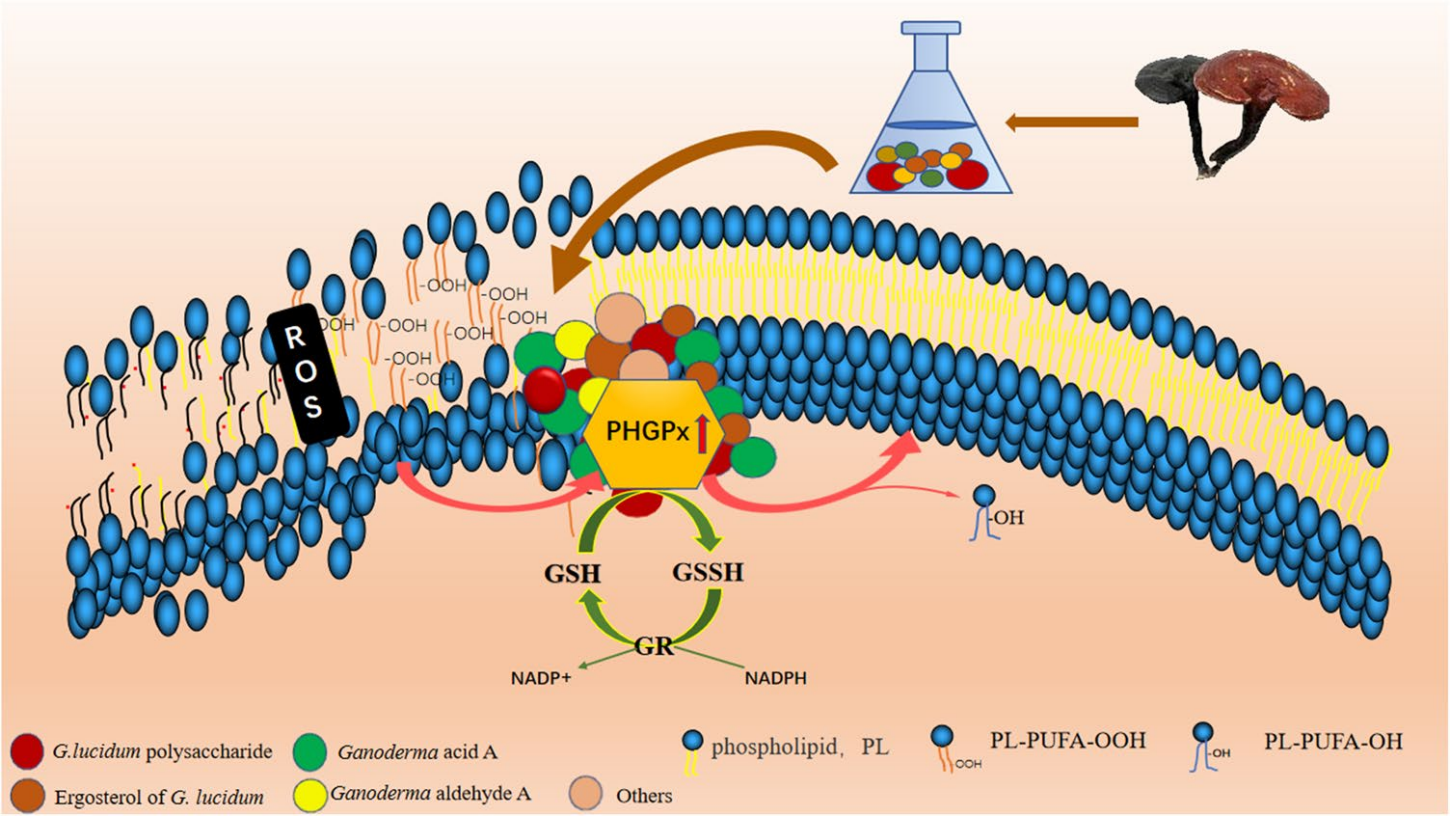
Schematic diagram of anti-aging mechanism of *G. lucidum* aqueous extract based on PHGPx.

In general, the *G. lucidum* aqueous extract promoted the expression of the selenium-dependent enzyme PHGPx in the GPX family. The enhancement of PHGPx activity could not only reduce the oxidative stress response in vivo but also specifically and effectively reduce the lipid deposition of biological membrane and maintain the integrity of the membrane (Fig. 7). Consequently, the active substances in the *G. lucidum* aqueous extract enter the cells through intact membrane and promote their metabolism. This created a virtuous cycle to achieve the effect of anti-aging.

## Supplementary Information

Supplementary Information.

## Data Availability

The authors declare that all other data supporting the findings of this study are available within the article and its supplementary information files.

## References

[1] Wang J, Cao B, Zhao HP, Feng J (2017). Emerging roles of *Ganoderma Lucidum* in anti-aging. Aging Dis..

[2] Phu HT (2020). Herbal medicine for slowing aging and aging-associated conditions: Efficacy, mechanisms, and safety. Curr. Vasc. Pharmacol..

[3] Das A (2021). Edible mushrooms as functional iIngredients for development of healthier and more sustainable muscle foods: A flexitarian approach. Molecules.

[4] Taufek N (2020). Performance of mycelial biomass and exopolysaccharide from Malaysian *Ganoderma lucidum* for the fungivore red hybrid Tilapia (*Oreochromis* sp.) in zebrafish embryo. Aquacult. Rep..

[5] Usuldin S (2021). In vivo toxicity of bioreactor-grown biomass and exopolysaccharides from Malaysian tiger milk mushroom mycelium for potential future health applications. Sci. Rep..

[6] Wan-Mohtar W, Ilham Z, Jamaludin AA, Rowan N (2021). Use of zebrafish Embryo assay to evaluate toxicity and safety of bioreactor-grown exopolysaccharides and endopolysaccharides from european Ganoderma applanatum Mycelium for future aquaculture applications. Int. J. Mol. Sci..

[7] Lin ZB (2019). *Ganoderma* (Lingzhi) in traditional Chinese medicine and Chinese culture. Adv. Exp. Med. Biol..

[8] Bishop KS (2015). From 2000 years of *Ganoderma lucidum* to recent developments in nutraceuticals. Phytochemistry.

[9] Seweryn E, Ziala A, Gamian A (2021). Health-Promoting of polysaccharides extracted from *Ganoderma lucidum*. Nutrients.

[10] Klupp NL, Kiat H, Bensoussan A, Steiner GZ, Chang DH (2016). A double-blind, randomised, placebo-controlled trial of *Ganoderma lucidum* for the treatment of cardiovascular risk factors of metabolic syndrome. Sci. Rep..

[11] Sen L (2020). Neural protective effects of millet and millet polyphenols on high-fat diet-induced oxidative stress in the brain. Plant Foods Hum. Nutr..

[12] Xu MM (2021). Influence of selenium biofortification on the growth and bioactive metabolites of *Ganoderma lucidum*. Foods..

[13] Tian R, Geng YP, Yang Y, Seim I, Yang G (2021). Oxidative stress drives divergent evolution of the glutathione peroxidase (GPX) gene family in mammals. Integr. Zool..

[14] Toppo S, Vanin S, Bosello V, Tosatto SC (2008). Evolutionary and structural insights into the multifaceted glutathione peroxidase (Gpx) superfamily. Antioxid Redox Signal..

[15] Deepalakshmi K, Mirunalini S, Krishnaveni M, Arulmozhi V (2013). In vitro and in vivo antioxidant potentials of an ethanolic extract of *Ganoderma lucidum* in rat mammary carcinogenesis. Chin. J. Nat. Med..

[16] Cech TR (1988). Ribozymes and their medical implications. JAMA.

[17] Blackburn EH (1989). Recognition and elongation of telomeres by telomerase. Genome.

[18] Collins K, Gorovsky MA (2005). *Tetrahymena thermophila*. Curr. Biol..

[19] López-Otín C, Blasco MA, Partridge L, Serrano M, Kroemer G (2013). The hallmarks of aging. Cell.

[20] Ferro D (2020). Molecular characterization protein-protein interaction network, and evolution of four glutathione peroxidases from *Tetrahymena thermophila*. Antioxidants (Basel)..

[21] Diaz S (2016). High resistance of *Tetrahymena thermophila* to paraquat: Mitochondrial alterations, oxidative stress and antioxidant genes expression. Chemosphere.

[22] Cubas-Gaona LL, de Francisco P, Martín-González A, Gutiérrez JC (2020). *Tetrahymena* glutathione peroxidase family: A comparative analysis of these antioxidant enzymes and differential gene expression to metals and oxidizing agents. Microorganisms..

[23] Li FX, Wang PF, Zhao C, Bao WY, Qiu LH (2017). Cloning and characterization of PHGPx and its synergistic role with p53 in mediating stress in *Penaeus monodon*. Fish Shellfish Immunol..

[24] Bain PA, Schuller KA (2012). A glutathione peroxidase 4 (GPx4) homologue from southern bluefin tuna is a secreted protein: First report of a secreted GPx4 isoform in vertebrates. Comp. Biochem. Physiol. B Biochem. Mol. Biol..

[25] Cuong VT (2019). The anti-oxidation and anti-aging effects of *Ganoderma lucidum* in *Caenorhabditis elegans*. Exp. Gerontol..

[26] Foissner W (2014). An update of 'basic light and scanning electron microscopic methods for taxonomic studies of ciliated protozoa. Int. J. Syst. Evol. Microbiol..

[27] Rastogi RP, Singh SP, Häder DP, Sinha RP (2010). Detection of reactive oxygen species (ROS) by the oxidant-sensing probe 2',7'-dichlorodihydrofluorescein diacetate in the cyanobacterium *Anabaena variabilis* PCC 7937. Biochem. Biophys. Res. Commun..

[28] Takemura G, Onodera T, Millard RW, Ashraf M (1993). Demonstration of hydroxyl radical and its role in hydrogen peroxide-induced myocardial injury: Hydroxyl radical dependent and independent mechanisms. Free Radic. Biol. Med..

[29] Nakagawa K, Kang SD, Park DK, Handelman GJ, Miyazawa T (1997). Inhibition of beta-carotene and astaxanthin of NADPH-dependent microsomal phospholipid peroxidation. J. Nutr. Sci. Vitaminol. (Tokyo).

[30] Fei WD (2020). Targeted GSH-exhausting and hydroxyl radical self-producing manganese-silica nanomissiles for MRI guided ferroptotic cancer therapy. Nanoscale.

[31] Chang WT, Gao ZH, Lo YC, Wu SN (2019). Evidence for effective inhibitory actions on hyperpolarization-activated cation current caused by Ganoderma Triterpenoids, the main active constitutents of *Ganoderma* Spores. Molecules.

[32] Bhardwaj A (2015). HPTLC based chemometrics of medicinal mushrooms. J. Liq. Chromatogr. Relat. Technol..

[33] Ha DT (2013). In vitro and in vivo hepatoprotective effect of ganodermanontriol against t-BHP-induced oxidative stress. J. Ethnopharmacol..

[34] Sharma P, Tulsawani R (2020). *Ganoderma lucidum* aqueous extract prevents hypobaric hypoxia induced memory deficit by modulating neurotransmission, neuroplasticity and maintaining redox homeostasis. Sci. Rep..

[35] Zhao C (2018). Pharmacological effects of natural Ganoderma and its extracts on neurological diseases: A comprehensive review. Int. J. Biol. Macromol..

[36] Xu J (2021). The versatile functions of *G. lucidum* polysaccharides and *G. lucidum* triterpenes in cancer radiotherapy and chemotherapy. Cancer Manag. Res..

[37] Gill BS, Navgeet MR, Kumar V, Kumar S (2018). Ganoderic acid, lanostanoid triterpene: A key player in apoptosis. Investig. New Drugs..

[38] Gao JJ (2002). New triterpene aldehydes, lucialdehydes A-C, from *Ganoderma lucidum* and their cytotoxicity against murine and human tumor cells. Chem. Pharm. Bull. (Tokyo).

[39] Lv GP, Zhao J, Duan JA, Tang YP, Li SP (2012). Comparison of sterols and fatty acids in two species of *Ganoderma*. Chem. Cent. J..

[40] Tadokoro T (2020). Mitochondria-dependent ferroptosis plays a pivotal role in doxorubicin cardiotoxicity. JCI Insight..

[41] Imai H (2003). Early embryonic lethality caused by targeted disruption of the mouse PHGPx gene. Biochem. Biophys. Res. Commun..

[42] Stradaioli G, Sylla L, Monaci M, Maiorino M (2009). Phospholipid hydroperoxide glutathione peroxidase in bull spermatozoa provides a unique marker in the quest for semen quality analysis. Theriogenology.

[43] de Almeida EA, Miyamoto S, Bainy AC, de Medeiros MH, Di Mascio P (2004). Protective effect of phospholipid hydroperoxide glutathione peroxidase (PHGPx) against lipid peroxidation in mussels *Perna perna* exposed to different metals. Mar. Pollut. Bull..

[44] Yang XD, Li WJ, Liu JY (2005). Isolation and characterization of a novel PHGPx gene in *Raphanus sativus*. Biochim. Biophys. Acta..

[45] Januel C (2006). Phospholipid-hydroperoxide glutathione peroxidase (GPx-4) localization in resting platelets, and compartmental change during platelet activation. Biochim. Biophys. Acta..

[46] Freitinger Skalická Z, Zölzer F, Beránek L, Racek J (2012). Indicators of oxidative stress after ionizing and/or non-ionizing radiation: superoxid dismutase and malondialdehyde. J. Photochem. Photobiol. B..

[47] Chai LH, Chen AX, Luo PO, Zhao HF, Wang HY (2017). Histopathological changes and lipid metabolism in the liver of *Bufo gargarizans* tadpoles exposed to Triclosan. Chemosphere.

