# Supplementary material for: Ganoderma lucidum aqueous extract inducing PHGPx to inhibite membrane lipid hydroperoxides and regulate oxidative stress based on single-cell animal transcriptome
Source: Sci Rep. 2022 Feb 24;12:3139. doi: 10.1038/s41598-022-06985-z (PMC8873301; doi:10.1038/s41598-022-06985-z)
Supplement: Supplementary file 1 — Supplementary Information. [file 41598_2022_6985_MOESM1_ESM.pdf]

Supplementary Information

Ganoderma lucidum aqueous extract inducing PHGPx to inhibite membrane lipid hydroperoxides and regulate oxidative stress based on single-cell animal transcriptome

Wenqiao Ding[1,2], Xueying Zhang[1], Xiaoyu Yin[1], Qing Zhang[1] Ying Wang[1], Changhong Guo[4] , Ying Chen[1,3*]

[1]Key Laboratory of Biodiversity of Aquatic Organisms, Harbin Normal University, Harbin,150025, China. [2]College of Biology and Food Engineering, Jilin Institute of Chemical Technology, Jilin,132022, China. [3]School of Civil and Environmental Engineering, Harbin Institute of Technology (Shenzhen), Shenzhen, 518055, China. [4]Key Laboratory of Molecular Cytogenetics and Genetic Breeding of Heilongjiang Province, College of Life Science and Technology, Harbin Normal University, Harbin, 150025, China.

Corresponding Author. *E-mail address:* chenying@hit.edu.cn

Methods

Real Time Quantitative PCR(RT-qPCR) RNA was extracted from the experimental group and control group by RNeasy protect cell Mini Kit (Qiagen). After the concentration and purity of RNA were determined, the corresponding RNA was obtained, and the cDNA was synthesized under the catalysis of reverse transcriptase. Samples of cDNA extracted from the control group and the experimental group were amplifiedand quadrupled in 96 microtiter plates (Applied Biosystems, Foster, CA, USA). Each PCR reaction (20 μL total volume) contained the following: 10 μL of SYBR Green PCR master mix 1×(Takara, Mou ntain View, CA, USA), 0.2 μM of each primer, and 5 μL of a 1/10 cDNA dilution. Three parallel samples were set for each reaction, and all the reactions were carried out on CFX96 real-time fluorescence quantitative PCR (Bio Rad, USA). The reaction conditions were 95 °C for 30 s, then 40 cycles of 10 s at 95 °C and then 20 s at 60 °C (3 biological replicates). Each group was subjected to fluorescent quantitative PCR with reference 18s. Primers were designed according to NCBI and TGD and synthesized by Shenggong biology Co., Ltd. and their quality was tested. The copy number of related genes was calculated by $2^{-\Delta\Delta Ct}$ method.

Determination of polysaccharide in *G. lucidum* aqueous extract Added 1 mL of *G.lucidum* aqueous extract with 4 times the volume of 90% ethanol, and stood at 4 °C overnight. Discard the supernatant, washed the sediment with 85% ethanol for several times, and then dissolved it measure. Suck 1mL of sample solution and measure the absorption according to the above standard curve steps.The content of polysaccharide in the aqueous extract reached 76%

Tables

Experiment number	*G. lucidum* (g)	*G. lucidum* polysaccharide (mg)	Water temperature (℃)	Time （h）	Concentration (%)
G1	1	0	50	2	80
G2	1.5	0	70	12	40

G3	2	0	90	1	20
G4	2.5	0	40	6	10
G5	3	0	60	0	5
G6	5	0	80	3	1
G7	10	0	100	24	100
A1	0	1	50	2	80
A2	0	1.5	70	12	40
A3	0	2	90	1	20
A4	0	2.5	40	6	10
A5	0	3	60	0	5
A6	0	3.5	80	3	1
A7	0	4	100	24	100

Table S1 Uniform design test plan for *G. lucidum* and *G. lucidum* polysaccharide.Number G1, G2, G3, G4, G5, G6 and G7 represent the experimental design of *G.lucidum* aqueous extract. Number A1, A2, A3, A4, A5, A6 and A7 represent the experimental design of *G.lucidum* polysaccharide.

Experiment number	*G. lucidum* monomer (mg)	Concentration (%)
B1/C1/D1	0.5	10
B2/C2/D2	1	80
B3/C3/D3	2	5
B4/C4/D4	5	40
B5/C5/D5	10	1
B6/C6/D6	20	20
B7/C7/D7	30	100

Table S2 Uniform design test plan for Ganoderma Acid A, Ganoderal A and G. lucidum ergosterol Number B1, B2, B3, B4, B5, B6 and B7 represent the experimental design of Ganoderic Acid A. Number C1, C2, C3, C4, C5, C6 and C7 represent the experimental design of Ganoderal A. Number D1, D2, D3, D4, D5, D6 and D7 represent the experimental design of *G. lucidum* ergosterol.

Number	Retention Time/min	Matching Degree/%	Compound	Type
1	6.235	96	Tetraethyl silicate	Esters
1	6.235	87	Ethyl stearate	Esters
2	7.561	98	1- Butanol	Alcohols
2	7.561	97	O-(1-methylethylethylidene)-D-mannitol	Alcohols
2	7.561	94	2- Heptanone	Aldehydes and ketones
2	7.561	94	4-methyl-3-pentene-2-one	Aldehydes and ketones
2	7.561	93	Octadeca-3,13-dien-1-ol	Alcohols
2	7.561	89	Hexadecan-1-ol	Alcohols
2	7.561	88	Docosan-1-ol	Alcohols
3	8.767	96	2- methyl- 1- Propano	Alcohols
3	8.767	85	Teramethylbenzene	Alkanes and derivatives
4	9.709	95	Pentamethylbenzene	Alkanes and derivatives
4	9.709	91	2-methylnaphthalene	Alkanes and derivatives
4	9.709	91	1-methylnaphthalene	Alkanes and

				derivatives
4	9.709	91	2,7-dimethylnaphthalene	Alkanes and derivatives
4	9.709	91	1,3-dimethylnaphthalene	Alkanes and derivatives
5	10.871	98	3- Octanone	Alcohols
5	10.871	98	3- methyl- 1- Butanol	Aldehydes and ketones
5	10.871	93	3,5-dimethyl-2-cyclohexen-1-one	Aldehydes and ketones
5	10.871	93	3-hexen-2-one	Aldehydes and ketones
6	14.132	96	2- Octanone	Alcohols
6	14.132	94	1- Hexanol	Alcohols
6	14.132	94	3- methyl- 6- methyleneoctane	Alkanes and derivatives
6	14.132	91	Glycerol	Alcohols
6	14.132	91	Butyl benzoate	Esters
6	14.132	87	Alpha-bulnesene	Alkanes and derivatives
6	14.132	85	Dodecamethyl- Cyclohexasiloxane	Alkanes and derivatives
7	18.532	98	Nonanoic acid	Acids
7	18.532	97	Formic acid, heptyl ester	Esters
7	18.532	97	Octadecanoic-9-enoicacid	Acids
7	18.532	97	Octadecanoic-6-enoicacid	Acids
7	18.532	97	Oleic acid	Acids
7	18.532	97	Acetic acid	Acids
7	18.532	95	1,4- dimethoxy- Benzene	Alkanes and derivatives
7	18.532	95	Hexanoic acid ethyl ester	Esters
8	21.032	98	Octanoic acid	Acids
8	21.032	98	3- Methylbutanoic acid	Acids
8	21.032	92	2,5- dimethylbenzophenone	Aldehydes and ketones
8	21.032	89	Benzoic acid	Acids
8	21.032	89	Stearic acid	Acids
8	21.032	89	Palmitic acid	Acids
8	21.032	95	(25R)-7β,15α-Dihydroxy-3,11,23-trioxo-5α-lanost-8-en-26-oic acid（Ganoderic Acid A）	Acids
9	24.316	96	4,5- di- epi- aristolochene	Alkanes and derivatives
9	24.316	96	Pentadecane	Alkanes and derivatives
9	24.316	93	1-benzyl-4-methylebnzene	Alkanes and derivatives
9	24.316	87	Eicosylbenzene	Alkanes and derivatives
9	24.316	87	3-methyl-1,1'-biphenyl	Alkanes and derivatives
9	24.316	85	1- bromo- 3- chloro- 2- methyl- Benzene	Other
10	26.132	96	Dilute acid	Acids
10	26.132	93	Formic acid, heptyl ester	Esters
10	26.132	90	1,1-dimethoxynonane	Alkanes and derivatives
10	26.132	94	2-Methyl-6-[(5R,10S,13R,14R,17S)-4,4,10,13,14-pentamethyl-3-oxo-1,2,5,6,12,15,16,17-octahydrocyclopenta[a]phenanthren-17-yl]hept-2-enal	Aldehydes and ketones
11	27.314	85	5,6-dihydroergosterol	Alcohols
11	27.314	92	Ergosta-4,6,8(14),22-tetraen-3-one	Aldehydes and ketones
11	27.314	92	7,22-ergostadienone	Aldehydes and ketones

Table S3 Chemical constituents of *G. lucidum* aqueous extract

Chemical Compound	Species	Relative Percentage Content/%
Alcohols	11	69.334

Aldehydes and ketones	8	6.749
Hydrocarbons and their derivatives	17	4.975
Acids	13	3.118
Lipids	5	0.954
Other Compound	1	0.078
Total	55	85.208

Table S4 Analysis of main components of *G. lucidum* aqueous extract

**Name**	**Sequence （5′-3′）**
18s	Forward: CCTGGGAAGGTACGGGTAAT<br>Reverse：AAGGTTCACCAGACCATTCG
SOD2	Forward: GGCAAGACACGGAACCAAAT<br>Reverse: CAACATCTCCCTAACCTTCTACTGG
CAT	Forward: GTTACCCATGACGTTACC<br>Reverse：GTAGAACTTGACTGCGAAAC
GPX1	Forward: CTTGGGCTGAGTCTGAAA<br>Reverse: GAAAAGAGGGAATTCTACGT
GPX2	Forward: CCCGATGGTAAGGTTCA<br>Reverse: CAGCTATCATATCGTTAGGAT
GPX3	Forward: GTGATGGTACAGTTCATAGTT<br>Reverse: AGGTTCAATTTCTTTTGGTT
GPX4	Forward: GTTGACTTCCCACTTTTCTCCA<br>Reverse: TGGGATCTTAGTTGCATAGTTGTTC
GST1	Forward: GGGGAATTTAAGCTTACTGA<br>Reverse: CAGCTCTTTTGTATTTCCTG
GST53	Forward: ATATTACTGCTACTAACGAACCT<br>Reverse: TCATATGGTACTTAGAGGAG
TrxR	Forward: TTTGCAATAGGAGATGCTGTT<br>Reverse: CCCTTATTTTATTGCCACAGG

Table S5 Primer sequences used in RT-qPCR

**Symbal name**	**Description**	**Logarithmic phase regulate**	**Decline phase regulate**
GPX 1	phospholipid hydroperoxide glutathione peroxidase	Up	Up
GPX2	phospholipid hydroperoxide glutathione peroxidase	Down	Up
GPX3	glutathione peroxidase	Up	No
GPX5	phospholipid hydroperoxide glutathione peroxidase	No	Up
GPX6	phospholipid hydroperoxide glutathione peroxidase	No	Up
GPX7	phospholipid hydroperoxide glutathione peroxidase	Up	Up
GPX8	phospholipid hydroperoxide glutathione peroxidase	No	Up
GPX9	phospholipid hydroperoxide glutathione peroxidase	Down	No
GPX10	predicted protein	Up	No
GPX11	phospholipid hydroperoxide glutathione peroxidase	Up	Up
GST53	glutathione S-transferase amine-terminal domain protein	Up	No
GST61	glutathione S-transferase amine-terminal domain protein	Up	No

Table S6 Analysis of genes up and down regulation in PPI network

Instrument name	Manufacturer
HH-8 digital display electrothermal constant temperature water bath pot	Changzhou Yitong analytical instrument manufacturing Co.,LTD
Circulating water multipurpose vacuum pump SHB-III	Shanghai Yukang science and education instrument equipment Co., Ltd
Tb-214 one ten thousandth electronic balance	Beijing saidoris Instrument System Co., Ltd
Allegra 64R desktop high-speed freezing centrifuge	Beckman Coulter, Inc.
Constant temperature shaker	Germany GFL3033
Fluorescence quantitative PCR	Bio-Rad
GCMS-QP2010	Shimadzu Corporation
Agilent 100 liquid chromatograph	Agilent Technologies Inc.
Transmission Electron Microscopy H-7650	Hitachi Limited
722 spectrophotometer	Shanghai Lengguang Technology Co., Ltd
-80 ℃ refrigerator	Midea Group

Table S7 Main instruments and equipment

Figures

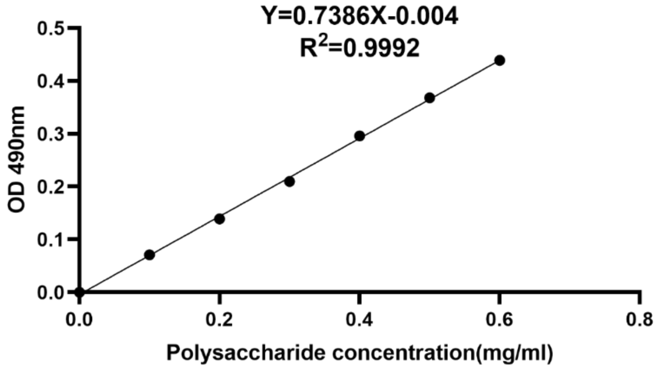

Figure S1. Polysaccharide standard curve

.

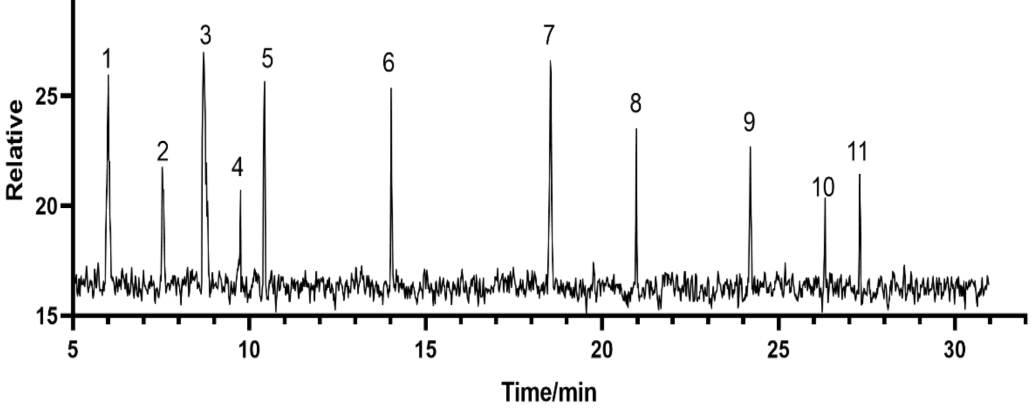

Figure S2. Total-ion flow chromatography of *G. lucidum* aqueous extract

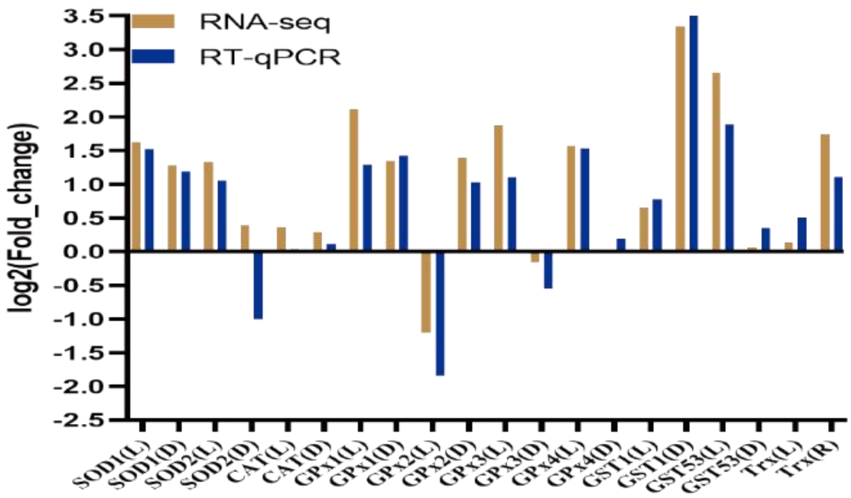

Figure S3. Confirmation of RNA-Seq data with RT-qPCR.

L,representative logarithmic phase; D, representative decline phase
